# Myoclonic movement after general anesthesia: A case report and review of the literature

**DOI:** 10.1097/MD.0000000000010141

**Published:** 2018-03-23

**Authors:** Je Jin Lee, Seho Lim, Yeon Sil Lee, Hwa-Yong Shin, Chong-Wha Baek, Yong Hun Jung, Young Cheol Woo, Yong-Hee Park

**Affiliations:** Department of Anesthesiology and Pain Medicine, Chung-Ang University Hospital, Seoul, Republic of Korea.

**Keywords:** epilepsies, general anesthesia, myoclonus, nefopam, partial, propofol

## Abstract

**Rationale:**

Myoclonic movement is a rare side effect after general anesthesia. Since we use various intravenous agents during general anesthesia recently, it is troublesome to find out the exact cause of this neurologic complication.

**Patient concerns:**

A 31-year-old female patient without any past medical history underwent hip arthroscopic surgery under general anesthesia.

**Diagnoses:**

Although there was no specific event during the operation, she showed a sudden myoclonic movement confined to left upper extremity in recovery room.

**Interventions:**

We administered anticonvulsant agents intrvenously, the myoclonus was stopped shortly but recurred over again. As we stopped the patient-controlled analgesia due to nausea, the symptom halted.

**Outcomes:**

There was no significant abnormality in electroencephalography or brain diffusion magnetic resonance imaging, which was taken after the event.

**Lessons:**

Clinicians should carefully consider the pharmacologic characteristics and neurologic adverse effects of all administered agents when myoclonus occurs after general anesthesia.

## 1 Introduction

In the recovery room, there are many problems observed. Some complications involve the cardiovascular system, such as hypotension and tachycardia, or the respiratory system, such as respiratory depression and upper airway obstruction. In addition, pain, postoperative nausea, vomiting, and shivering are commonly observed. In terms of neurologic problems, there are mostly reports of emergence agitation, delirium, or postoperative cognitive disorder. Notably, myoclonic movements or seizures are not common findings in the immediate postoperative period. However, there have been some reports that show a relationship between the different agents used as anesthetics (e.g., propofol)^[1]^ or drugs for perioperative management (e.g., ramosetron or nefopam)^[2,3]^ and neurologic disorders (especially myoclonic movement). We observed a case of myoclonic movement in the recovery room after the patient received general anesthesia for an elective orthopedic surgery.

## 2 Case presentation

A 31-year-old female patient was scheduled for elective hip arthroscopic surgery for osteoarthritis. She was 157 cm tall, 48 kg in weight (BMI 19.5), and had neither a family medical history or own past medical history of the disease. She was not taking any medication routinely before surgery. All results from preoperative laboratory tests including an electrocardiogram, a chest x-ray, as well as blood and urine examinations were in the normal range.

The patient entered the operation room without any premedication. We began monitoring noninvasive blood pressure, electrocardiogram activity, oxygen saturation, and bispectral index (BIS). General anesthesia was induced with fentanyl 100 μg, 2% lidocaine 40 mg, and 1% propofol 80 mg via intravenous injection. Rocuronium 35 mg was injected immediately following loss of consciousness. We used a plain endotracheal tube (internal diameter of 7 mm) for endotracheal intubation after confirming that neuromuscular junction blockade was completed. During the operation, inhalation of 4% to 6 vol% of desflurane (with 50% oxygen and 50% nitrous oxide) was used for the maintenance of anesthesia and the depth of anesthesia was controlled to maintain a BIS level between 40 and 60. The end tidal carbon dioxide tension was in the range of 35–37 mm Hg.

We applied a patient-controlled analgesia (PCA) pump using fentanyl 1000 μg, nefopam 80 mg, and ramosetron 0.3 mg at the time of initiation of surgical suturing. The total volume of fluid in PCA was 100 mL, which included a bolus volume of 1.0 mL, followed by delivery at a basal flow rate of 1.0 mL/h. The lockout time for PCA was 15 minutes. At the end of the operation, the neuromuscular blockade was reversed by intravenous administration of 100 mg sugammadex. The patient was extubated after she recovered consciousness and could obey commands. The operation lasted for 50 minutes (operation time) and the patient was under anesthesia was 103 minutes (anesthetic time).

After arriving in the recovery room, the patient's vital signs were stable. Oxygen was supplied at a flow rate of 5 L/min via a simple facial mask. Her mental status changed from drowsy to alert after 10 minutes of arriving in the recovery room. At this time, she suddenly complained of a myoclonic jerk, which lasted for 10 minutes, involving the upper part of her left arm. There were no observations of salivation, urination, or biting of the tongue. The patient's orientation remained intact and she was able to obey verbal commands. The patient asserted that she could not control her left arm at the location of the involuntary movement.

Just after the myoclonic movement was observed, 2 mg of midazolam was administered via intravenous injection and she recovered immediately. Thirty minutes later, when she awoke from a sedative state, the same myoclonic movement recurred. Thiopental 50 mg was administered which again stopped the myoclonic movement. A third attack (in the same manner as the previous two) occurred 45 minutes after her arrival to the recovery room. A neurologist physically examined the patient, which did not reveal any abnormal findings except for the involuntary movement of her left shoulder and upper arm. According to the neurologist, the condition could be diagnosed as a partial seizure. As such, he ordered to start continuous intravenous infusion of valproate 1 g mixed with 100 mL of normal saline for an hour. The patient was transferred to the general ward and administered oxygen 2 L/min via nasal cannula. In the general ward, she complained of nausea. Therefore, the PCA was clamped and no other antiemetic agents were administered. The myoclonic movement of her left arm was abolished thereafter.

After the patient had been in the general ward for 2 hours, she underwent an electroencephalography (EEG) for neurologic evaluation. Furthermore, after 7 hours, she underwent a brain diffusion magnetic resonance imaging to acquire T2/FLAIR images. There were no significant abnormalities. Valproate 400 mg mixed with 100 mL of normal saline was administered from the day after the operation for a duration of 12 hours until she was discharged. Cefotetan 1 g was injected twice a day for antibiotic prophylaxis. The myoclonus did not recur during hospitalization and the patient was discharged on the third day after operation without sequelae. No other medications except analgesics were given. Three months after the event, she did not report any symptoms at the outpatient clinic. This case's Naranjo Scale is 2 so we suspect it as a possible adverse drug reaction.

## 3 Discussion

Myoclonic movement after general anesthesia is not common event. However, there were several case reports that presented myoclonus either in postanesthetic care unit during recovery phase or delayed onset in ward or even after discharge.^[1,2,4–6]^ Propofol, fentanyl, ramonsetron, and nefopam were drugs that were suspicious of the cause of postoperative myoclonic movement in those previous cases.

Propofol is widely used to induce general anesthesia. It is a preferred medication as both an anesthetic and sedative due to its characteristics of rapid onset, short acting, and brief recovering time. Commonly reported side effects of propofol are hypotension, respiratory depression, and local intravascular pain at the site of injection. Reported neurological complications are uncommon, but do include unconsciousness, altered mental status, myoclonic movement, opisthotonus, ataxia, seizure-like movements, and twitching movements.^[7]^ In particular, there are few existing case reports of myoclonic movements, which were observed postanesthesia with delayed onset.^[1,8]^ Most of propofol-induced myoclonus went with alteration of the patient's mental status, but there is a case report that a patient underwent involuntary myoclonic movements with unaltered mental status.^[4]^ The mechanisms of the seizure-like movements after administration of propofol are still relatively unknown. However, there is a possible relationship between myoclonic movements and the effect of propofol on the gamma aminobutyric acid (GABA) pathway. In GABA pathway, a balance is lost between the activity of excitatory and inhibitory neurons at high tissue-concentrations of propofol (in the manner of a drug-induced delayed elimination).^[9]^ However, in this particular case, we need to examine the effects of the other anesthetic agents involved.

In this present case, fentanyl and ramosetron were combined with nefopam in the PCA. High-dose fentanyl (200–400 μg/kg) has been shown to induce sharp waves with an epileptic pattern in the EEG of a rat model.^[10]^ There are also a few case reports that describe grand mal seizures after fentanyl administration in humans.^[11,12]^ For example, a 79-year-old woman without an unusual medical history developed grand mal seizures when 200 μg of fentanyl was administered in divided doses during the induction of general anesthesia.^[11]^ However, there were no seizure-like movements observed in our patient when fentanyl was administered during the induction of general anesthesia, and the dose of fentanyl in the PCA (10 μg/mL) was very low compared with that of the previous cases. In addition, these cases were characterized as each patient underwent grand mal seizures while our patient had a myoclonus with unaltered mental status.

Ramosetron is a serotonin (5-hydroxytryptamine type 3 receptor [5-HT3 R]) antagonist used for its antiemetic effect. Recently, physicians prefer the use of 5-HT3 R antagonists because, among the antiemetic agents, they do not have side effects such as sedation or extrapyramidal symptoms. Seizure is a rare side effect of 5-HT3 R antagonists but there have been some reported cases.^[2,5]^ In these cases, the exact cause of the seizures was not identified; however, by the process of exclusion of other drugs, the 5-HT3 R antagonists were considered to have caused the condition. In most cases of 5-HT3 R antagonist-induced seizures, the drug was administered when the patients were conscious. However, in our case, we administered ramosetron when the patient was anesthetized and there was a long-time gap before the occurrence of the first seizure in the recovery room. As such, a causal relationship between the 2 events cannot be derived.

Nefopam (Acupan, Pharmbio Korea, Chungju, Korea), a class of benzoxacines, is a centrally acting nonopioid drug used for the prevention of shivering or relief from acute or chronic pain. Adverse effects of nefopam such as confusion, hallucinations, convulsions, dizziness, nausea, vomiting, tachycardia, and palpitations have been reported. The use of nefopam is contraindicated in patients with convulsive disorders because of 7 reported cases of generalized tonic-clonic seizures after nefopam administration. Neuropsychiatric or cardiovascular adverse effects are mostly observed in patients administered an overdose of nefopam. In cases of generalized tonic-clonic seizure followed by fatality, the serum concentration of nefopam was in the range of 4.3 to 11.9 mg/L.^[13,14]^ There has also been a case report of status epilepticus occurring in a patient who was treated with a continuous infusion of nefopam.^[6]^ In this case, the patient was treated with various pain relievers because of back pain, and 80 mg of nefopam was mixed with 1 L of plasma solution and administered at an infusion rate of 42 mL/h. The typical dosage for the intravenous form of nefopam is 10 to 20 mg by slow injection every 4 to 6 hours or 10 to 30 mg by intravenous infusion.^[15]^ A report from the French Pharmacovigilance database also commented that serious, neurological, adverse drug reactions could occur within the range of appropriate therapeutic dosage.^[3]^ In this case, a low dosage of nefopam was administered to our patient. Nefopam was infused via PCA at the rate of 0.8 mg/h and, if the bolus button was pushed, the maximum dose of nefopam infused in one hour was 4.0 mg. However, the patient was very sensitive to the PCA medications and her myoclonic movement ceased when the PCA was clamped. For this reason, we doubt that nefopam may be the cause of the myoclonus. Unlike previous studies reporting that general seizures could be a side effect of nefopam, our patient had a possible partial seizure limited to her left arm. In previous cases, there were usually reports of confusion or convulsions, as well as one report of a petit mal seizure in a 65-year-old female patient who took nefopam at 60 mg per day for 5 days.

## 4 Conclusion

In conclusion, myoclonic movement can occur in the recovery room, even following uneventful general anesthesia in patients without risk factors. From the review of previous reports about postioperative myoclonus, drugs that are commonly used in routine anesthetic procedures such as propofol, fentanyl, ramosetron, and nefopam can cause myoclonic movements or seizures even in small doses. Therefore, clinicians should carefully consider the pharmacologic characteristics and neurologic adverse effects of all administered agents.

## 5 Authors’ contributions

JJL made substantial contributions to conception and design of the case review; SL, YSL, and HYS. have been involved in drafting the manuscript or revising it critically for important intellectual content; YHJ gave final approval of the version to be published; CHB and YCW made substantial contribution to conception, design, and interpretation of the case review; YHP, was accountable for all aspects of the work in ensuring that questions related to the accuracy or integrity of any part of the work are appropriately investigated and resolved.
